# The role of mitochondrial labile iron in Friedreich's ataxia skin fibroblasts sensitivity to ultraviolet A†
†Electronic supplementary information (ESI) available. See DOI: 10.1039/c8mt00257f


**DOI:** 10.1039/c8mt00257f

**Published:** 2019-02-19

**Authors:** Olivier Reelfs, Vincenzo Abbate, Agostino Cilibrizzi, Mark A. Pook, Robert C. Hider, Charareh Pourzand

**Affiliations:** a Department of Pharmacy and Pharmacology , University of Bath , Claverton Down , Bath BA2 7AY , UK . Email: C.A.Pourzand@bath.ac.uk; b Institute of Pharmaceutical Science , King's College London , Franklin-Wilkins Building, 150 Stamford Street , London SE1 9NH , UK; c King's Forensics , Department of Analytical , Environmental and Forensic Sciences , School of Population Health & Environmental Sciences , King's College London , 150 Stamford Street , London SE1 9NH , UK; d Division of Biosciences , Brunel University London , Kingston Lane , Uxbridge , UB8 3PH , UK

## Abstract

Mitochondrial labile iron (LI) is a major contributor to the susceptibility of skin fibroblasts to ultraviolet A (UVA)-induced oxidative damage leading to necrotic cell death *via* ATP depletion. Mitochondria iron overload is a key feature of the neurodegenerative disease Friedreich's ataxia (FRDA). Here we show that cultured primary skin fibroblasts from FRDA patients are 4 to 10-fold more sensitive to UVA-induced death than their healthy counterparts. We demonstrate that FRDA cells display higher levels of mitochondrial LI (up to 6-fold on average compared to healthy counterparts) and show higher increase in mitochondrial reactive oxygen species (ROS) generation after UVA irradiation (up to 2-fold on average), consistent with their differential sensitivity to UVA. Pre-treatment of the FRDA cells with a bespoke mitochondrial iron chelator fully abrogates the UVA-mediated cell death and reduces UVA-induced damage to mitochondrial membrane and the resulting ATP depletion by a factor of 2. Our results reveal a link between FRDA as a disease of mitochondrial iron overload and sensitivity to UVA of skin fibroblasts. Our findings suggest that the high levels of mitochondrial LI in FRDA cells which contribute to high levels of mitochondrial ROS production after UVA irradiation are likely to play a crucial role in the marked sensitivity of these cells to UVA-induced oxidative damage. This study may have implications not only for FRDA but also for other diseases of mitochondrial iron overload, with the view to develop topical mitochondria-targeted iron chelators as skin photoprotective agents.

## Abstract 2

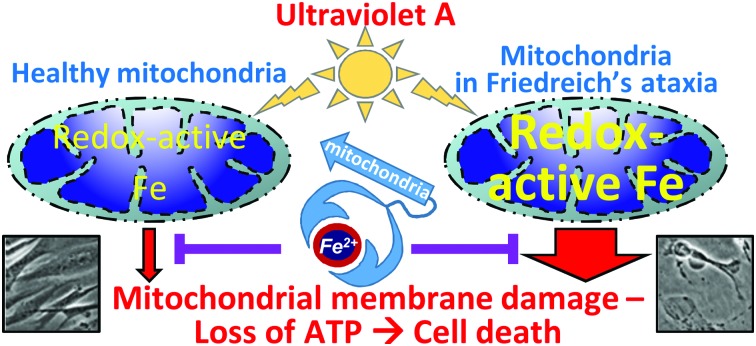
Friendreich's ataxia skin cells are highly sensitive to ultraviolet A due to their high levels of mitochondrial redox-active iron.

##

Significance to metallomics

Iron is a vital element for cell processes such as mitochondrial energy production. In Friedreich's ataxia (FRDA), dysregulation of iron metabolism causes mitochondrial iron overload, leading to excess reactive oxygen species production and cytotoxicity. We report that high levels of mitochondrial iron in skin cells from FRDA patients render them extremely sensitive to oxidative stress induced by solar ultraviolet A (UVA) compared to their healthy counterparts. Moreover, a bespoke mitochondrial iron-trapping molecule protects the FRDA-affected skin cells from UVA-induced damage. This study highlights the sensitivity to UVA as a novel connection to FRDA and possibly other mitochondrial iron overload disorders.

## Introduction

Iron is an indispensable element for life as it participates in several crucial cellular functions.^1^ However, iron can also be potentially cytotoxic when present in the form of redox-active chelatable labile iron (LI) which can act as catalyst in the formation of harmful reactive oxygen species (ROS) such as hydroxyl radical *via* Fenton chemistry.^2^ Hence, levels of LI are normally tightly regulated in cells. The majority of the intracellular LI resides in subcellular compartments, with mitochondria, the main cellular site of iron metabolism having been shown to be the major destination of LI.^3^–^5^ Due to their function in respiration, mitochondria are also the primary source of ROS in cells, hence the presence of high chelatable LI in mitochondria renders these organelles particularly susceptible to oxidative stress conditions. We have previously demonstrated that mitochondria are a major target of UVA radiation-induced damage in skin fibroblasts leading to ATP depletion and the ensuing necrotic cell death.^6^–^9^ Subsequently we showed that targeting the mitochondrial LI of skin fibroblasts with a highly specific chelator provides an unprecedented protection against UVA-mediated oxidative damage to the organelle and the resulting cell death.^10^

In view of the central role of mitochondria in cells, it is not surprising that deregulation of mitochondrial iron metabolism, such as that which occurs in Friedreich's ataxia (FRDA), has profound consequences on cell integrity and function. FRDA is a disease caused by deficient levels of frataxin (FXN), a mitochondrial protein^11^ which plays a key role as an iron chaperone in the synthesis of heme and iron sulfur clusters (ISCs) and also in antioxidant protection.^12^,^13^ A major long-term consequence of pathological or genetically-mediated reduction of FXN levels in cells and tissues is mitochondrial iron overload which has been linked to progressive mitochondria dysfunction, impairment of energy metabolism and accumulation of intracellular oxidative damage.^14^–^17^ Furthermore, cultured skin fibroblasts from FRDA patients show defects in antioxidant mechanisms^18^–^20^ and are more sensitive than their healthy counterparts to various forms of chemically-induced oxidative stress,^21^ including exogenous iron and H_2_O_2_.^22^,^23^ Both iron- and H_2_O_2_-induced cytotoxicity can be partially reversed when cells are pre-treated with iron chelators, implying an important contribution from mitochondrial LI in oxidative injury. Treatment of a mouse model of FRDA with highly lipophilic iron chelators mitigated the symptoms of the disease on the heart, further supporting the benefits of this approach.^24^ Furthermore, treatment of cells with deferiprone (DFP), a membrane-permeable bidentate iron chelator reduced oxidative damage to mitochondrial proteins.^25^,^26^ Several clinical trials have demonstrated the therapeutic benefits of DFP – used alone or in combination with other molecules, in the treatment of FRDA.^26^–^28^ Clearly the use of iron chelators to reinstate mitochondria functions and cell/tissue integrity in FRDA has potential. Nevertheless, all the chelators used so far systemically – including DFP, have drawbacks related to their lack of target specificity for the site of damage (*i.e.* mitochondria) and the consequent risks including disruption of normal homeostasis and depletion of patient's iron levels, resulting in anaemia. Hence there is a clear need to design iron chelators with improved properties, such as targeting them to their site of action, the mitochondria.

One approach to reduce/avoid unwanted effects and improve the effectiveness of the chelator consists in targeting the chelating unit to the mitochondria by linking it to a mitochondria-specific “SS peptide”. Such peptides, based on alternate hydrophobic and basic amino acid side chains readily permeate cell plasma membranes and selectively accumulate in the mitochondria.^29^–^32^ We have recently developed and characterized two distinct classes of fluorescent bidentate iron chelators selectively directed to mitochondria and acting as iron sensors.^29^,^33^–^37^ Furthermore, we demonstrated the successful use of a hexadentate mitochondria-targeted iron chelator in photoprotection of cultured skin fibroblasts against solar UVA (320–400 nm)-induced mitochondria damage and ensuing cell death.^10^

Because mitochondrial iron overload has been recognised as a key feature of FRDA,^38^,^39^ we hypothesized that FRDA patients might be more susceptible to UVA-induced skin damage than healthy counterparts. Here we show that skin fibroblasts from FRDA patients are highly sensitive to UVA-induced cytotoxicity compared to their healthy counterparts. This correlated with the higher basal level of mitochondrial LI in FRDA cells which contributes to higher LI-catalysed ROS generation in the organelle following UVA irradiation. We further demonstrate that pre-treatment of human fibroblasts prior to UVA irradiation with a mitochondria-targeted iron chelator suppresses the LI-catalysed ROS production and cell death and halves both the damage to mitochondrial membrane and ATP depletion in the human derived FRDA fibroblasts. Similar results were also observed in fibroblast cells derived from a mouse model of FRDA. Our study describes a novel effect of mitochondrial dysregulation and suggests a link between the high levels of both mitochondrial redox active LI and ROS in FRDA skin fibroblasts and their particular sensitivity to UVA. To our knowledge this study is the first demonstration of this link. These findings further suggest that topical use of mitochondria-targeted iron chelators may be effective for skin photoprotection of FRDA and possibly other disorders of mitochondria iron overload exhibiting skin photosensitivity to sunlight.

## Materials and methods

### Iron chelator–peptide synthesis

The design, synthesis and characterization of compound **2** (“cpd **2**”) and compound **13** (“cpd **13**”), the chelator–peptides used in this study have been documented elsewhere.^10^,^29^ Both of these compounds have been previously shown to accumulate selectively to the mitochondrial compartment, using quantitative fluorescence microscopy.^10^,^29^

### Reagents

All reagents used for cell cultivation were obtained from Life Technologies (Paisley, Scotland). Phosphate buffered saline (PBS) was from Oxoid Ltd (Basingstoke, UK). The mitochondrial markers tetramethylrhodamine methyl ester (TMRM) and the mitochondrial ROS indicator mitoSOX™ red were purchased from Invitrogen (Paisley, Scotland). Annexin V-FLUOS was from Roche (Welwyn Garden City, UK). All other reagents were from Sigma-Aldrich (Gillingham, UK).

### Cell culture

The human primary skin fibroblasts GM04078 (male, 30 years old), GM03816 (female, 36 years old) and GM03665 (female, 12 years old) from clinically affected Friedreich's ataxia patients and fibroblasts AG09429 (female, 25 years old) from an apparently healthy (control) donor were obtained from the Coriell Institute for Medical Research (Camden, New Jersey). These lines have been characterized elsewhere notably with respect to their relative levels of FXN.^40^,^41^ Relative levels of FXN protein: FRDA GM03816 (medium-low) > GM04078 (low) > GM03665 (very low). The human primary skin fibroblasts FEK4 which were used as a control line (courtesy of Prof. R. Tyrrell's laboratory, Switzerland) were isolated and expanded from tissue samples obtained in full compliance with ethical regulations and legislation in force at the time and respecting complete donor confidentiality.^10^ FEK4 cells were isolated from foreskin biopsies (passage 0), then subcultured and frozen at increasing passages (up to p12). These stocks were available for the present study. Two mouse fibroblast cell lines derived from a transgenic mouse model of FRDA were also used: Y47R (from control mouse) expresses normal level of FXN and YG8sR (derived from “FRDA-like” mouse) expresses significantly reduced level of FXN.^42^

The human primary fibroblast cells were routinely grown in Earle's MEM supplemented with 15% FCS, glutamine and antibiotics, at 37 °C in a 5% CO_2_ atmosphere, as described previously.^10^ The mouse cells were grown in DMEM supplemented with 10% FCS and antibiotics.^42^,^43^ For experiments involving either chelator–peptide and/or mitoSOX delivery, phenol red and antibiotics were omitted from the medium.

### Compound delivery

The iron chelator–peptides (cpd **2** and cpd **13**) were prepared as 100 mM stock solutions in DMSO (≥99.7% purity, cell culture grade), diluted to the final desired concentration of 50 μM in conditioned medium and added to cells overnight as described previously.^10^,^29^ Final DMSO concentration in the medium during treatment never exceeded 0.1%. On the day following treatment, cells were irradiated where required and further incubated in conditioned medium without compound for the periods indicated.

### Determination of mitochondrial labile iron

The principle and protocol of the determination of mitochondrial LI are described in Supplementary Materials and methods and in Fig. S2 (ESI†).

### Determination of mitochondrial ROS production

Cells were grown in 6 cm plates in duplicates for three days to reach 70–80% confluency. On day 2 of the seeding, cpd **2** was added to the corresponding plates at a final concentration of 50 μM. Next day, cells were washed with PBS and either irradiated with a UVA dose of 250 kJ m^–2^ or sham-irradiated (*i.e.* control). Immediately following treatment, cells were incubated in serum-free and phenol red-free EMEM containing 1.5 μM mitoSOX™ red for 30 min at 37 °C. After two washes with PBS, cells were collected by trypsinization and resuspended in PBS containing 0.1% BSA. Cells were then counted and triplicates of each condition, containing equal numbers of cells (0.1–0.2 × 10^6^ cells) were analysed using a spectrofluorimeter Clariostar (BMG LABTECH Ltd, Aylesbury, UK) using PBS–0.1% BSA as blank. Red fluorescence was detected using 451–522 nm bandwidth for excitation and 550–650 nm bandwidth for emission, for broad detection of mitochondrial ROS.^44^

### UVA irradiation

Irradiations were performed in PBS at approximately 25 °C using a broad spectrum Sellas 4 kW UVA lamp (Sellas, Germany), as described previously.^45^ The incident dose rate was *ca.* 0.2 kJ m^–2^ s^–1^. UV doses were measured using an IL1700 radiometer (International Light, Newbury, Massachusetts). Control cells were treated in the same manner to irradiated ones, except that they were not irradiated (*i.e.* sham-irradiated).

### MTT assay

Cells grown in 35 mm dishes were treated as indicated in the figure legends and grown for a further 24 h in conditioned medium, after which the latter was removed and the cells were incubated for 3 h at 37 °C in serum-free medium containing 0.5 mg mL^–1^ MTT (3-(4,5-dimethylthiazol-2-yl)-2,5-diphenyl-tetrazolium bromide). MTT solution was then removed and DMSO was added to the cells in order to dissolve the formazan formed. Absorbance was read at 550 nm using a spectrophotometer Fluostar Optima (BMG LABTECH Ltd, Aylesbury, UK). The percentage of viable cells was calculated by comparing the absorbance of treated *versus* non-treated control cells.

### Flow cytometry

All flow cytometry protocols are described in Supplementary Materials and methods (ESI†).

### ATP measurement

ATP production by cells following various treatments was monitored with a luminometer (Turner Designs Model TD-20/20) 2 h after UVA, using the ViaLight™ plus kit (Lonza Biologics plc, Slough, UK) as described previously.^46^

### Live cell microscopy

Bright-field images used to document morphological changes following treatments were captured on a Motic inverted microscope (model AE2000) (Motic Deutschland GmbH, Wetzlar, Germany) *via* a Moticam 580 digital camera and a Plan 10× objective.

### Statistical methods

Results are expressed as the mean ± standard deviation (SD). Significant differences (*P* < 0.05) were determined by either paired or unpaired *t*-test after one-way analysis of variance.

## Results

### Fibroblasts isolated from Friedreich's ataxia patients show acute photosensitivity to biologically relevant doses of UVA

**Fig. 1:**
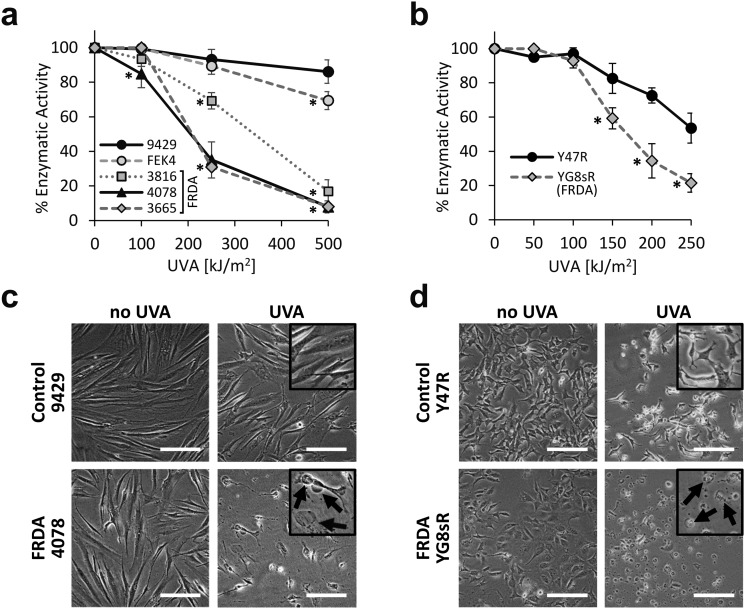
FRDA fibroblasts are significantly more sensitive to UVA than their healthy counterparts. Cells were irradiated with increasing doses of UVA radiation and cell metabolic activity was assessed by MTT 24 h post-treatment. Dose–response curves are shown for the human fibroblasts (a) and for the mouse fibroblasts (b). Curves are representative of *n* = 3–8 independent experiments in (a) and *n* = 3–6 in (b). * significantly different (*p* < 0.05) from control cell lines at corresponding dose. (c) Bright-field images were captured 24 h following irradiation of human fibroblasts with 250 kJ m^–2^ UVA or (d) mouse fibroblasts with 150 kJ m^–2^ UVA. Cellular damage (arrows in inset) in the form of cell swelling and cell debris is visible after UVA irradiation. Scale bar: 50 μm.

We first investigated the sensitivity of primary fibroblast cell cultures derived from healthy patients and from patients diagnosed with FRDA, to a range of physiologically and environmentally relevant UVA doses. The MTT assay was used as a measurement of viable cell metabolism. Primary skin fibroblasts from FRDA patients are deficient in FXN and have been shown to provide a suitable model of the signature molecular characteristics of the disease.^19^ The cell lines were challenged with increasing doses of UVA ranging from 100 to 500 kJ m^–2^ (*ca.* 30 min to 2.6 h exposure to sunlight at sea level^47^) and the MTT assay was performed 24 h post-irradiation (Fig. 1a). At a dose as low as 100 kJ m^–2^, the sensitivity of the FRDA lines GM03816 and GM04078 was *ca.* 10% higher than that of control cell lines. At a dose of 250 kJ m^–2^ (*i.e.* equivalent to *ca.* 77 min exposure to sunlight at sea level) all three FRDA cell lines displayed an increasingly higher sensitivity than their healthy counterparts, with GM04078 and GM03665 being *ca.* 3-fold more sensitive. At the highest dose of 500 kJ m^–2^ (equivalent to *ca.* 154 min sunlight at sea level), the sensitivity of the FRDA lines rose up to 10-fold over that of cells from healthy donors. Our data show that FRDA skin cells are more sensitive than their healthy counterparts to physiologically relevant doses of UVA. Microscopic images of control (AG09429) and FRDA (GM04078) cells irradiated at 250 kJ m^–2^ UVA capture the morphological changes 24 h post-treatment (Fig. 1c). Cell swelling, indicative of death by necrosis is visible in the FRDA cells, whereas control cells appear essentially unaffected.

We then investigated whether the differential sensitivity to UVA observed in the human fibroblasts also held true in cells derived from a mouse model of FRDA.^42^ In this model, FRDA mouse (YG8sR) carries the FXN transgene from an FRDA patient, and thus expresses low levels of FXN compared to control mouse (Y47R) which expresses the FXN transgene from an unaffected donor. YG8sR and Y47R fibroblasts were irradiated at UVA doses from 50 up to 250 kJ m^–2^ and cell viability was assessed by MTT 24 h post-irradiation (Fig. 1b). YG8sR cells were more sensitive than control Y47R cells in the UVA dose range from 150 up to 250 kJ m^–2^, displaying up to 2-fold difference in viability. Microscopic images of cells irradiated at a UVA dose of 150 kJ m^–2^ document the morphological changes 24 h post-treatment (Fig. 1d).

### A mitochondria-targeted iron chelator rescues fibroblasts isolated from Friedreich's ataxia patients from UVA-induced cell death

**Fig. 2:**
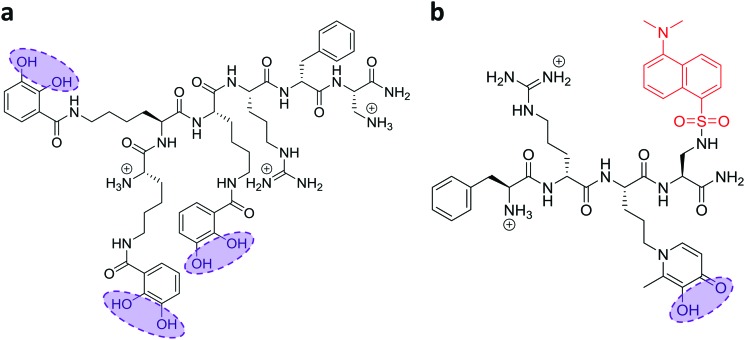
Structures of the mitochondrial iron chelator–peptides (“compounds **2** and **13**”). The structures of the compounds used in this study are depicted with their ionized functions at pH 7.4. (a) The three catechol functions (circled) form the iron-binding moieties which together bind iron as a hexadentate chelator for compound **2**. (b) Likewise, the hydroxypyridinone chelating moiety in compound **13** is circled and the fluorescent probe dansyl is coloured in red.

**Fig. 3:**
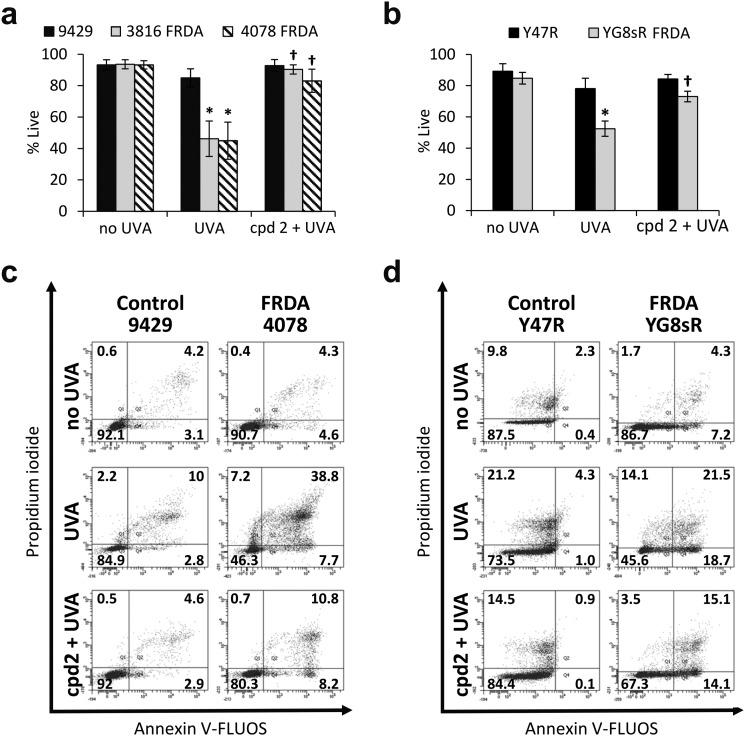
The chelator–peptide (compound **2**) fully protects FRDA fibroblasts against UVA-induced cell death. Human fibroblasts (a) and mouse fibroblasts (b) were treated (or not) overnight with 50 μM compound **2**, then irradiated with UVA and the percentage of live cells was determined 24 h post-treatment by flow cytometric Annexin V/PI double-labelling. UVA doses used: 500 kJ m^–2^ for human cells, 250 kJ m^–2^ for mouse cells. Results from 3–6 independent experiments are expressed as means ± standard deviation (SD) of percentage live cells. * significantly different (*p* < 0.05) from irradiated control cells. † significantly different (*p* < 0.05) from corresponding sample irradiated with UVA alone. Representative dot-plots shown for human fibroblasts (c) and for mouse fibroblasts (d).

In recent work, we reported the effectiveness of a mitochondria-targeted iron chelator peptide, cpd **2** (Fig. 2a) in protecting human skin fibroblasts in culture against UVA-induced mitochondrial damage, loss of ATP and cell death.^10^ The chimeric compound which is based on a mitochondria-tropic “SS peptide” carries three iron-chelating moieties, circled in dotted lines. We sought to investigate as a first step whether cpd **2** would be able to protect cells from FRDA patients from UVA-induced cell death. For this purpose, cells were pre-treated (or not) with the compound as indicated in Materials and methods, then irradiated with a UVA dose of 500 kJ m^–2^ and live and dead/dying cells were scored 24 h following irradiation using flow cytometry and dual labelling of cells with fluorescently-labelled Annexin V and propidium iodide (PI). This technique allows to determine whether cells are viable, apoptotic or necrotic *via* differences in plasma membrane integrity and permeability. Percentages of live cells obtained are plotted as bar charts in Fig. 3a for the FRDA cell lines GM03816 and GM04078 using AG09429 as control. Non-irradiated cells from all three cell lines display similar levels of live cells (90–95%). Upon irradiation (500 kJ m^–2^), the population of live AG09429 cells decreased by *ca.* 10%. In stark contrast, irradiated FRDA GM03816 and GM04078 cells incurred a significant drop of 50–60% of viable cells compared to non-irradiated sample. However, pre-treatment with cpd **2** prior to irradiation effectively restored viability of control AG09429 cells, but also of the two FRDA fibroblasts to the level of untreated sample. Visual inspection of microscopy images of these experiments (Fig. S1a, ESI†) highlights the morphological changes indicative of cell death in FRDA cells following UVA irradiation alone, and the robust protection afforded by cpd **2**. Representative dot plots of the results obtained for AG09429 and GM04078 are shown (Fig. 3c), in which live cells occupy the lower left-hand side quadrant (negative for both dyes) of each graph.

We then sought to interrogate the responsiveness of the mouse cell lines Y47R and YG8sR to cpd **2** following UVA insult. In view of the observed higher sensitivity of mouse fibroblasts (*ca.* 2–2.5-fold) to UVA compared to human fibroblasts (see Fig. 1), we chose the lower dose of 250 kJ m^–2^ (approximately equitoxic to 500 kJ m^–2^ in human FRDA fibroblasts) to carry out our investigation and cells were pre-treated (or not) with cpd **2** as in Fig. 3a prior to irradiation. As can be seen in Fig. 3b, the irradiation treatment caused a drop of 10% live cells in Y47R cells, whereas the FRDA YG8sR cells sustained a much greater loss of *ca.* 40% live cells. Pre-treatment of cells with cpd **2** prior to irradiation restored the percentage live cells to its value in untreated sample in Y47R and to *ca.* 85% of its value in untreated sample in YG8sR. Representative dot plots from these experiments are depicted in Fig. 3d. Microscopy images of these experiments (Fig. S1b, ESI†) document the marked morphological changes undergone by the FRDA cells following UVA irradiation and their disappearance in cells pre-treated with cpd **2**. Taken together, these data show that cpd **2** is capable of effectively protecting highly UVA-sensitive FRDA cells, from both patients and a mouse model of FRDA against UVA-induced death.

### Skin fibroblasts from patients with FRDA have higher basal mitochondrial LI and UVA-induced ROS levels than their healthy counterparts

**Fig. 4:**
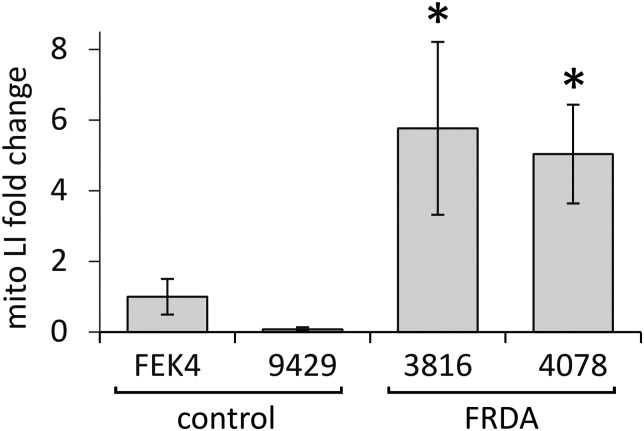
Mitochondria from FRDA fibroblasts have significantly higher levels of labile iron compared to healthy fibroblasts. Cells were treated (or not) with the hexadentate chelator DFO followed by 50 μM of compound 13 as described in materials and methods. Cells were then harvested and their fluorescence was measured. Data were compiled from *n* = 3–5 measurements per cell line. Mitochondrial LI levels are represented as fold change relating to control FEK4 cells (taken as 1). * significantly different (*p* < 0.05) from healthy cells.

**Fig. 5:**
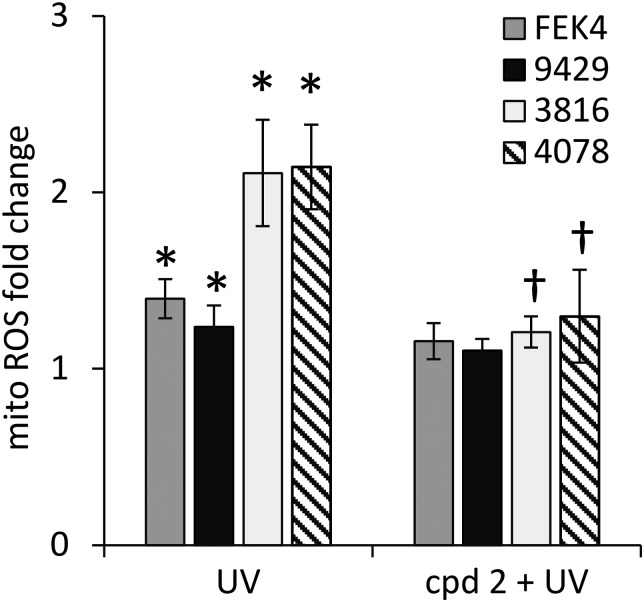
Mitochondria from FRDA fibroblasts have higher levels of ROS compared to healthy fibroblasts. Cells were treated (or not) with cpd **2**, then with a UVA dose of 250 kJ m^–2^ and analysed using the mitochondrial probe mitoSOX red as described in materials and methods. Data were compiled from *n* = 3–4 measurements per cell line. Mitochondrial ROS levels are indicated as fold change relating to their corresponding unirradiated control cells (taken as 1). * significantly different (*p* < 0.05) from corresponding unirradiated control cells. † significantly different (*p* < 0.05) from corresponding sample irradiated with UVA alone.

In order to gain further understanding of the role of mitochondrial LI in the higher phototoxicity of UVA to skin fibroblasts from patients with FRDA, we sought to measure the level of mitochondrial LI in the cell lines used in this study. For this purpose, we used a sensitive mitochondria-targeted iron-selective “turn-off” fluorescent sensor – cpd **13** (Fig. 2b and Fig. S2, ESI†), which we previously developed and characterized in human primary fibroblasts.^29^ cpd **13**, which carries a hydroxypyridinone-based function can selectively sense variations in intracellular concentrations of mitochondrial LI. Our results (Fig. 4) show that the mean levels of mitochondrial LI in FRDA cells GM03816 and GM04078 (1.11 μM ± 0.37) are on average 6.5-fold higher than those in cells from healthy donors FEK4 and AG09429 (0.17 μM ± 0.12). In order to assess if the higher level of mitochondrial LI in FRDA cells was correlated with increased oxidative stress, we measured the levels of mitochondrial ROS by spectrofluorimetry in cells exposed (or not) to UVA-mediated oxidative stress. For this purpose, we used the mitochondrial fluorescent probe mitoSOX, whose fluorescence increases following oxidation by ROS.^44^ Cells, either non-irradiated or irradiated with a UVA dose of 250 kJ m^–2^ were analysed immediately following treatment for changes in mitochondrial ROS, as described in Materials and methods (Fig. 5). UVA irradiation treatment increased the levels of mitochondrial ROS production over background by a factor of *ca.* 2.1 on average in FRDA cell lines and by 1.2-fold on average in healthy cell lines. This represents an average increase of 1.8-fold of mitochondrial ROS production in the FRDA cells studied compared to healthy counterparts. Preincubation of cells with cpd **2** prior to UVA irradiation efficiently prevented the UVA-induced mitochondrial ROS production increase in cells from both healthy and FRDA donors.

### Mitochondria of fibroblasts from patients with Friedreich's ataxia are markedly more sensitive to UVA compared to those from their healthy counterparts

**Fig. 6:**
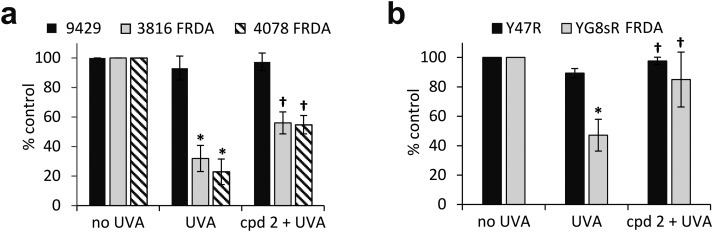
UVA-induced damage to the mitochondria of FRDA cells is efficiently reduced by the mitochondrial iron chelator. Human fibroblasts (a) and mouse fibroblasts (b) were treated (or not) overnight with 50 μM compound **2**, then irradiated with UVA and the magnitude of mitochondria membrane depolarisation was measured 2 h post-treatment by flow cytometry using TMRM labelling. UVA doses: 250 kJ m^–2^ for human cells, 150 kJ m^–2^ for mouse cells. Results from 3–6 independent experiments are expressed as means ± SD of percentage fluorescence compared to untreated control cells. * significantly different (*p* < 0.05) from irradiated control cells. † significantly different (*p* < 0.05) from corresponding sample irradiated with UVA alone.

The pathological accumulation of iron in the mitochondria of patients with FRDA is thought to be a strong contributor to the observed enhanced sensitivity to oxidative stress of the cells of those patients. We hypothesized that this mechanism could exacerbate the sensitivity of mitochondria from FRDA patients to damage induced by environmental solar UVA and that this could potentially be counteracted by the use of a mitochondria-specific iron chelator. Mitochondria functionality can be assessed by the integrity of mitochondria membrane potential (Δ*ψ*_m_) using flow cytometry and the fluorescent cationic dye tetramethylrhodamine methyl ester (TMRM) which accumulates only in intact mitochondria. Mitochondrial membrane depolarization translates into a decrease in TMRM fluorescence. Human healthy and FRDA fibroblasts were irradiated with a UVA dose of 250 kJ m^–2^ and fluorescence was monitored 2 h post-irradiation, a time-point at which we have previously shown in healthy fibroblasts that both the level of labile iron in the cell and damage to membranes are at their highest.^6^–^8^ The UVA dose of 250 kJ m^–2^ was chosen so that the resulting membrane damage would not lead to significant dye leakage, which would invalidate the assay. The results, reported as a bar chart in Fig. 6a, are expressed as percentage fluorescence of non-irradiated cells (*i.e.* control). In healthy AG09429 cells, irradiation caused a non-significant drop in TMRM fluorescence of *ca.* 10% compared to control cells. In contrast, in both FRDA cell lines GM03816 and GM04078, a significant decrease of respectively 70 and 80% in TMRM fluorescence could be observed, indicative of the exacerbated sensitivity of mitochondria. Pre-treatment of FRDA cells with cpd **2** prior to irradiation afforded a significant 2-fold and 3-fold protection to GM03816 and GM04078 cells, respectively while healthy AG09429 cells fully recovered their mitochondria integrity. We then interrogated the mouse fibroblasts similarly to the human fibroblasts above but using the UVA dose of 150 kJ m^–2^ (approximately equitoxic to 250 kJ m^–2^ in human FRDA fibroblasts). The FRDA YG8sR cells showed much higher sensitivity to UVA irradiation when compared to their healthy counterparts Y47R, with a *ca.* 50% drop in TMRM fluorescence compared to 10% in Y47R (Fig. 6b). When pre-treated with compound **2** prior to irradiation, YG8sR cells displayed a 2-fold recovery of fluorescence signal, indicating significant mitochondria membrane protection, while Y47R cells fully recovered their mitochondrial membrane potential.

**Fig. 7:**
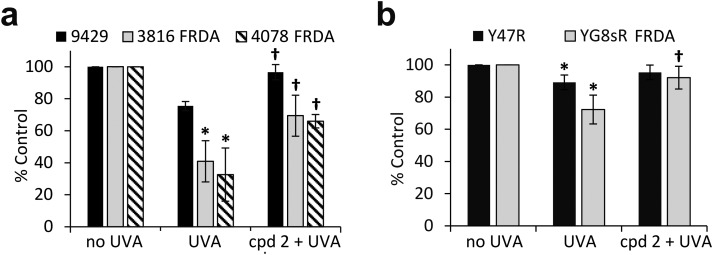
The mitochondrial iron chelator mitigates the UVA-induced loss of ATP in FRDA fibroblasts. Human fibroblasts (a) and mouse fibroblasts (b) were treated as in Fig. 6 and cellular ATP production was measured 2 h post-treatment. Results from 3–5 independent experiments are expressed as means ± SD of percentage ATP production compared to untreated control cells. * significantly different (*p* < 0.05) from irradiated control cells. † significantly different (*p* < 0.05) from corresponding sample irradiated with UVA alone.

We have previously shown that the measurement of cellular ATP is a good indicator of mitochondria integrity following exposure to biologically relevant doses of UVA radiation.^6^ We therefore sought to assess whether the observed high sensitivity of mitochondria of FRDA cells to UVA correlated with the levels of cellular ATP. Cells were treated under the same conditions as those employed to assess mitochondria integrity using TMRM, and ATP levels were measured 2 h post-irradiation (Fig. 7a). UVA irradiation of AG09429 cells caused a reduction in ATP levels of *ca.* 25% compared to non-irradiated controls, while ATP levels in irradiated FRDA cells were reduced by up to 70%. Pre-treatment of cells with cpd **2** prior to irradiation afforded a *ca.* 30% recovery of ATP levels in both FRDA GM03816 and GM04078 cells, while a full rescue of ATP levels in AG09429 was recorded. We then interrogated Y47R and YG8sR mouse cells as in the corresponding TMRM experiments (Fig. 7b). While ATP levels in Y47R cells were reduced by 10% following UVA irradiation, they were decreased by 30% in FRDA YG8sR cells. Significantly, pre-treatment of both Y47R and YG8sR cells with cpd **2** prior to irradiation almost fully restored ATP levels. In summary, these data demonstrate that mitochondria of fibroblasts from both FRDA patients and a mouse model of the disease are more sensitive to UVA-induced damage than their healthy counterparts, as measured by loss of mitochondrial membrane potential (*ψ*_m_) and decreased ATP production. Furthermore, we show that both mitochondria integrity and functionality can be protected by pre-treatment of cells with a mitochondria-targeted iron chelator.

## Discussion

Our findings reveal an important link between FRDA as a disease of mitochondrial iron overload and skin fibroblast sensitivity to UVA. Taken together, these results provide a link between the higher levels of mitochondrial LIP in FRDA cells and the higher oxidative stress observed in these organelles. These results are also in line with the observed much higher sensitivity of FRDA fibroblasts to UVA-induced damage compared to healthy counterparts. A high concentration of resident redox-active mitochondrial iron provides a rational explanation to the high oxidative burden in FRDA mitochondria described by others as a result of defective expression of frataxin.^12^ Such burden would be exacerbated upon exposure of cells to UVA radiation. The complete protection afforded by cpd **2** against UVA-induced cell death supports the crucial role of mitochondrial LI in this process. We suggest that the level of mitochondrial LI could be used a prognostic indicator for skin photosensitivity, with particular relevance for FRDA patients but also, for patients with other disease of mitochondrial iron overload.

Furthermore, we show that both mitochondria integrity and functionality can be protected, at least in part by pre-treatment of cells with a mitochondria-targeted iron chelator. Exposure of cells to cumulative biologically relevant doses of UVA has been shown to induce damage to mitochondrial DNA^48^ with concurrent effect on cell viability. Oxidation of sensitive mitochondrial proteins such as those containing ISCs can also occur as a result of excessive oxidative stress catalysed by excess mitochondrial LI, thereby impairing mitochondrial functions. Taken together our results are in favour of the concept that mitochondrial iron overload in FRDA skin cells will exacerbate UVA-induced ROS-mediated damage to mitochondrial components, especially ISC-containing proteins, resulting in compromised energy production, loss of mitochondrial function and cell death. The altered antioxidant response in FRDA cells^18^,^19^ will almost certainly be an important contributing factor in their greater sensitivity to UVA, the oxidizing component of sunlight. However, our data suggest that mitochondrial iron is the determining factor.

The high sensitivity of FRDA fibroblasts to the oxidative solar UVA waveband has practical implications. Despite lying deep in the skin, fibroblasts are a major target for skin photodamage by long-wave UVA since up to 50% of this UV range penetrates into the dermis.^49^ UVA directly affects the architecture of the dermis *via* the increase in matrix metalloproteinases activity, resulting in remodelling of the extracellular matrix through degradation of collagens and elastin which are hallmarks of photoaged skin.^50^,^51^ Our findings highlight the possibility that other skin cell sensitization may occur in other diseases characterised by mitochondria iron overload. In view of this, topical application of mitochondria-targeted iron chelators such as cpd **2** as part of a sunscreen formulation could be envisioned as a valuable approach for skin photoprotection of FRDA patients.

Finally, the results obtained in the mouse fibroblast model of FRDA recapitulate the trend in differential sensitivities observed in human fibroblasts between healthy and FRDA cells, albeit to a lesser degree of intensity, thereby broadening the validity of this study model for the field of FRDA research.

## Conclusion

We have shown that skin fibroblasts from FRDA patients are highly sensitive to exposure to UVA. Our results altogether tie in with the concept that mitochondrial redox active iron overload is a crucial component of this sensitivity due to its ability to participate in Fenton chemistry, generating highly damaging ROS in UVA-irradiated FRDA fibroblasts.^52^ The knowledge inferred from our study has relevance for healthcare professionals in prevention of sun-induced skin damage (including photoaging) and could function as a valuable early cautionary indicator to forewarn patients with FRDA (estimated prevalence of about 1 in 29 000 in Caucasians with a concentration of 1 in 20 000 in south-west Europe^53^,^54^). This may potentially extend to other diseases of mitochondrial iron overload. Skin fibroblasts which can readily be obtained from FRDA patients could be used as a predictive tool to determine their sensitivity to UVA. Furthermore, they represent a useful model to test photoprotective compounds including iron chelators, antioxidant molecules and sunscreen ingredients. Finally, we present the first application of our bespoke mitochondrial iron-selective sensor to measure mitochondrial redox-active iron levels in healthy and FRDA skin fibroblasts. With this unique new tool, this study is contributing to the development and validation of novel techniques aimed at the measurement of iron in specific organelles, with particular relevance to pathological conditions.

## Conflicts of interest

The authors state no conflict of interest.

## Supplementary Material

Supplementary information
