# Supplementary material for: The role of mitochondrial labile iron in Friedreich's ataxia skin fibroblasts sensitivity to ultraviolet A
Source: Metallomics. 2019 Feb 19;11(3):656–65. doi: 10.1039/c8mt00257f (PMC6438355; doi:10.1039/c8mt00257f)

Electronic Supplementary Material (ESI) for Metallomics.
This journal is © The Royal Society of Chemistry 2019

Supporting Information (ESI) for

The role of mitochondrial labile iron in Friedreich's ataxia skin fibroblasts sensitivity to ultraviolet A

Olivier Reelfs[1]*, Vincenzo Abbate[2]*, Agostino Cilibrizzi[2], Mark A. Pook[3], Robert C. Hider[2] and Charareh Pourzand[1**]

[1] Department of Pharmacy and Pharmacology, University of Bath, Claverton Down, Bath BA2 7AY, United Kingdom.
[2 a] Institute of Pharmaceutical Science, King's College London, Franklin-Wilkins Building, 150 Stamford Street, London SE1 9NH and [b] King's Forensics, Department of Analytical, Environmental and Forensic Sciences, School of Population Health & Environmental Sciences, King's College London, 150 Stamford Street, London SE1 9NH, United Kingdom.
[3] Division of Biosciences, Brunel University London, Kingston Lane, Uxbridge, UB8 3PH, United Kingdom.

* These authors contributed equally.

**Corresponding author: Charareh Pourzand, Department of Pharmacy and Pharmacology, University of Bath, Claverton Down, Bath BA2 7AY, United Kingdom.
Email: C.A.Pourzand@bath.ac.uk

SUPPLEMENTARY MATERIALS AND METHODS

Determination of mitochondrial labile iron

Briefly, 80% confluent cells were pretreated (or not) for 6-8 h with 100 μM of the hexadentate chelator desferrioxamine (DFO), with the purpose to deplete maximally the intracellular LI. Cells were then washed twice with serum-free medium and incubated overnight at 37 °C with 50 μM of the fluorescent iron sensor (compound **13**). The next day, cells were harvested by trypsinization, resuspended in buffer F (containing 10 mM HEPES, pH 7.3 and 150 mM NaCl) and counted. Equal number of cells were transferred to a thermostatically controlled (37 °C) quartz cuvette and kept in suspension for fluorescence reading (λexc =330 nm and λem = 550 nm) with a Hitachi F-4500 spectrofluorimeter (Lambda Advanced Technology Ltd, Uxbridge, UK). The difference in fluorescence between the two samples was taken as a measure of the concentration of the mitochondrial LI, which was quantified using an *ex situ* calibration curve. PBS, buffer F and the quartz cuvette were all chelex-treated so as to avoid unwanted quenching of compound's fluorescence.

Flow cytometry

All flow cytometry data were obtained using a Becton Dickinson FACSAria III. Cells were gated via forward scatter versus side scatter dot plot so as to exclude very small debris in the origin. Data were collected from a minimum of 10'000 cells (events) and analyzed with FACSDiva Software version 8.0.1 (Becton Dickinson, San Jose, CA). Dual staining with Annexin V/PI was carried out as described previously,[1] 24 h post-UVA irradiation. Cell populations were defined by quadrant analysis of dot plots of Annexin V-FLUOS versus PI and expressed as percentage. "Live" cells were defined as Annexin V-negative/PI-negative.

Double-positive and Annexin V-negative/PI-positive cells were considered “necrotic” and Annexin V-positive/PI-negative cells as “apoptotic’’. Single-labelled cell samples were used for quadrant setting and compensation. The mitochondrial transmembrane potential ($\Delta\psi_m$) was measured using TMRM labelling as described previously.[1] Briefly, the conditioned medium was removed 2 h following UVA treatment, cells rinsed with PBS and incubated for 30 min at 37 °C with 50 nM TMRM in phenol-red free Hank's Balanced Salt Solution (HBSS) supplemented with 10 mM 4-(2-hydroxyethyl)-1-piperazineethanesulfonic acid (HEPES). The cells were then collected by trypsinization, resuspended in HBSS buffer and median fluorescence intensities (MFI) scored.

SUPPLEMENTARY REFERENCE

1 O. Reelfs, V. Abbate, R. C. Hider and C. Pourzand, *J. Invest. Dermatol.* 2016, **136**, 1692-1700.

SUPPLEMENTARY FIGURES

Fig. S1 The chelator-peptide fully protects FRDA fibroblasts against UVA-induced cell death. Bright-field microscopic images were taken of cells treated in the conditions described in Fig. 3, immediately prior to performing the Annexin V/PI assay. Cell death by necrosis, characterized by cell swelling (indicated by arrows in the insets) is visible after UVA treatment alone of either human (a) or mouse fibroblasts (b). Note the disappearance of these signs in the images of cells which were pre-treated with the mitochondrial iron chelator prior to UVA irradiation. Scale bar 50 μm.

a

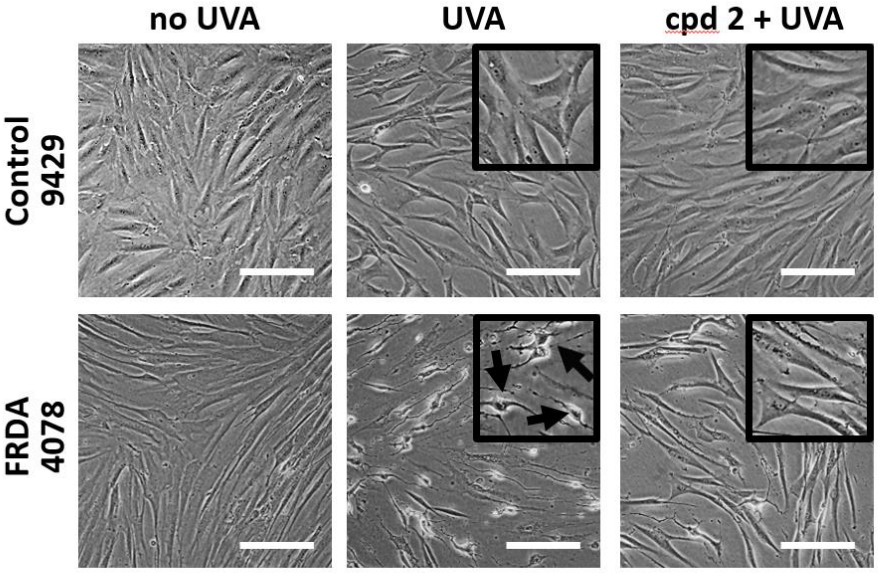

b

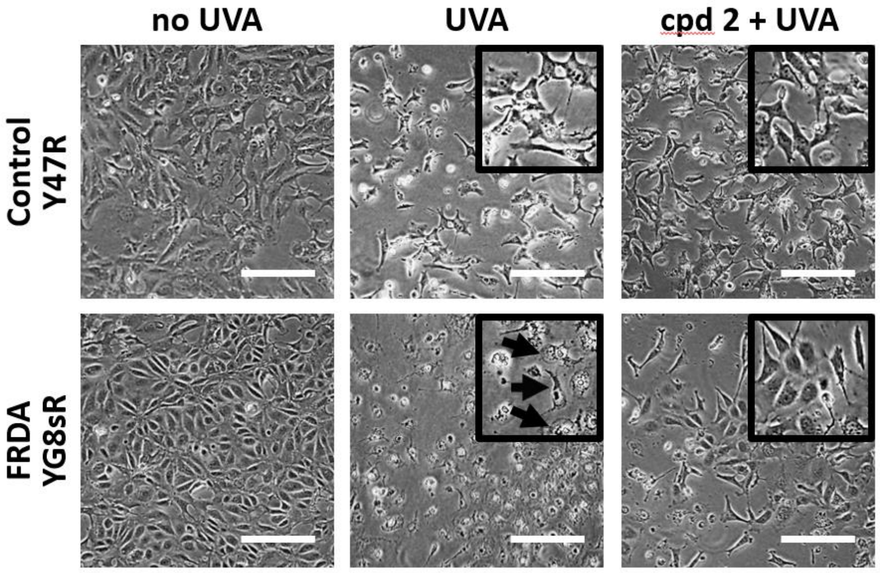

Fig. S2 Principle of determination of mitochondrial labile iron with compound **13** using spectrofluorimetry. (a) The determination of the mitochondrial LI is based on the (reversible) "quenching" of the fluorescence of compound **13** in its target compartment, upon binding to resident iron to form a stable complex (fluorescence F1). The maximum fluorescence (F2) of "unbound" compound **13** is revealed by treatment of cells with an excess (100 µM) of the strong iron chelator Desferrioxamine (DFO). Fluorescence "dequenching" (or Δ F) is proportional to the concentration of mitochondrial LI. (b) Δ F can then be converted to actual LI concentrations using an appropriate *ex-situ* calibration curve.

a

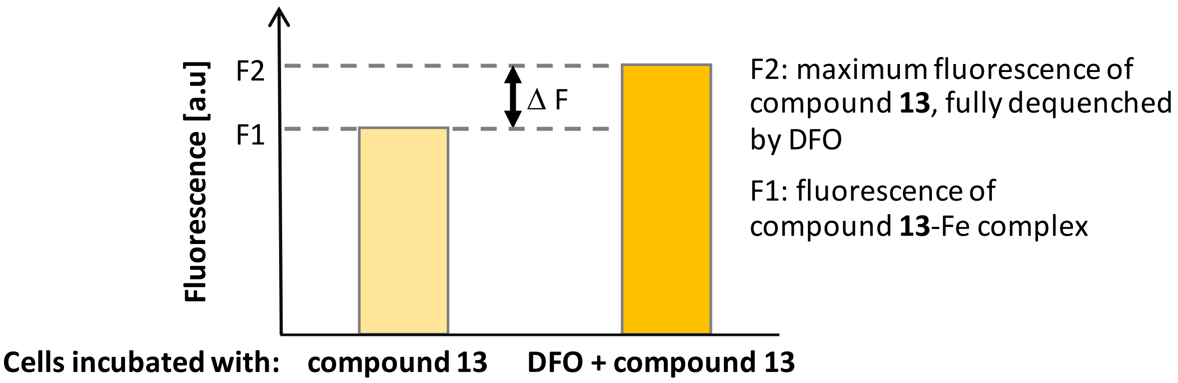

b

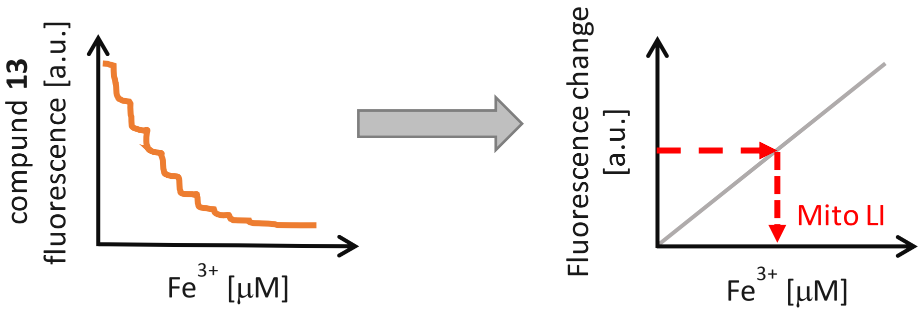

Supplement: Supplementary file 1 [file MT-011-C8MT00257F-s001.pdf]
